# Molecular dynamics simulation dataset of a kinesin on tubulin heterodimers in electric field

**DOI:** 10.1016/j.dib.2023.109765

**Published:** 2023-11-04

**Authors:** Jiří Průša, Michal Cifra

**Affiliations:** Institute of Photonics and Electronics of the Czech Academy of Sciences, Prague, 18200, Czechia

**Keywords:** Molecular dynamics, Electric field, Proteins, Microtubule, Kinesin

## Abstract

We present trajectories from non-equilibrium (in electric field) molecular dynamics (MD) simulations of a kinesin motor domain on tubulin heterodimers with two tubulin heterodimers forming neighbouring microtubule protofilaments. The trajectories are for no field (long equilibrium simulation), for four different electric field orientations (X, -X, Y, -Y) and for the X electric field at four different field strengths. We also provide a trajectory for larger simulation box. Our data enable to analyze the electric field effects on kinesin, which ultimately leads to kinesin detachment. This data set was used to understand the effect of electric field orientation and field strength on the kinetics and energetics of the electro-detachment of kinesin [1].

Specifications Table

**Table:** 

Subject	Biophysics, Physical Sciences, Engineering, Physical and Theoretical Chemistry
Specific subject area	Computational Molecular biophysics
Type of data	Molecular Dynamics (MD) simulations
How data were acquired	Classical all-atom MD computational simulation in explicit solvent
Data format	Raw: Compressed GROMACS trajectory (.xtc)
Parameters for data collection	NVT ensemble at 300 K
Description of data collection	Data were obtained from molecular dynamics simulations ran on CESNET MetaCentrum virtual organisation of the Czech National Grid Organization with GROMACS software version 5.1.1
Data source location	Institution: Institute of Photonics and Electronics of the Czech Academy of Sciences City/Town/Region: Prague Country: Czechia
Data accessibility	Data is stored in a public repository Repository name: ASEP Repository - Repository of Czech Academy of Sciences https://asep.lib.cas.cz/ Data identification number: 0572510 Direct URL to data: https://doi.org/10.57680/asep.0572510
Related research article	Jiří Průša, Michal Cifra, Electro-detachment of kinesin nanomotor from microtubule in silico, Computational and Structural Biotechnology Journal, 2003 DOI:10.1016/j.csbj.2023.01.018

## 1 Value of the Data

• Data enable understanding of the influence of intense nano-second scale electric field on the interaction of kinesin nanomotor and its microtubule track.
• Computational chemists, physical chemists, chemical biologists, bioelectromagnetic engineers and biophysicists can benefit from the data.
• The data can be further used for a further analysis of the structural and dynamics effects of electric field on kinesin nanomotor at the atomic level.

## 2 Objective

Intense pulsed electric field is capable of influencing cell membranes causing electropermeabilization and electroporation [2] having rapidly expanding biomedical and industrial applications [3], [4], [5]. However, much less is known about the effects of intense pulsed electric field on other biological structures such as proteins [6], [7], [8]. A great deal of understanding of the mechanism of action of intense electric field on biomolecules and proteins comes from molecular dynamics simulations [9], [10], [11], [12]. To understand better the effects of intense electric field on cytoskeletal proteins, we generated a molecular dynamics simulation of kinesin motor domain on a segment of microtubule exploring several electric field strength values and field vector directions. The full data set enables further analysis and provides a more detailed description of the simulation settings beyond the data in the original research article [1].

## 3 Data Description

The raw data (440 GB) are available in archive files under this permanent link:

https://doi.org/10.57680/asep.0572510

**Fig. 1:**
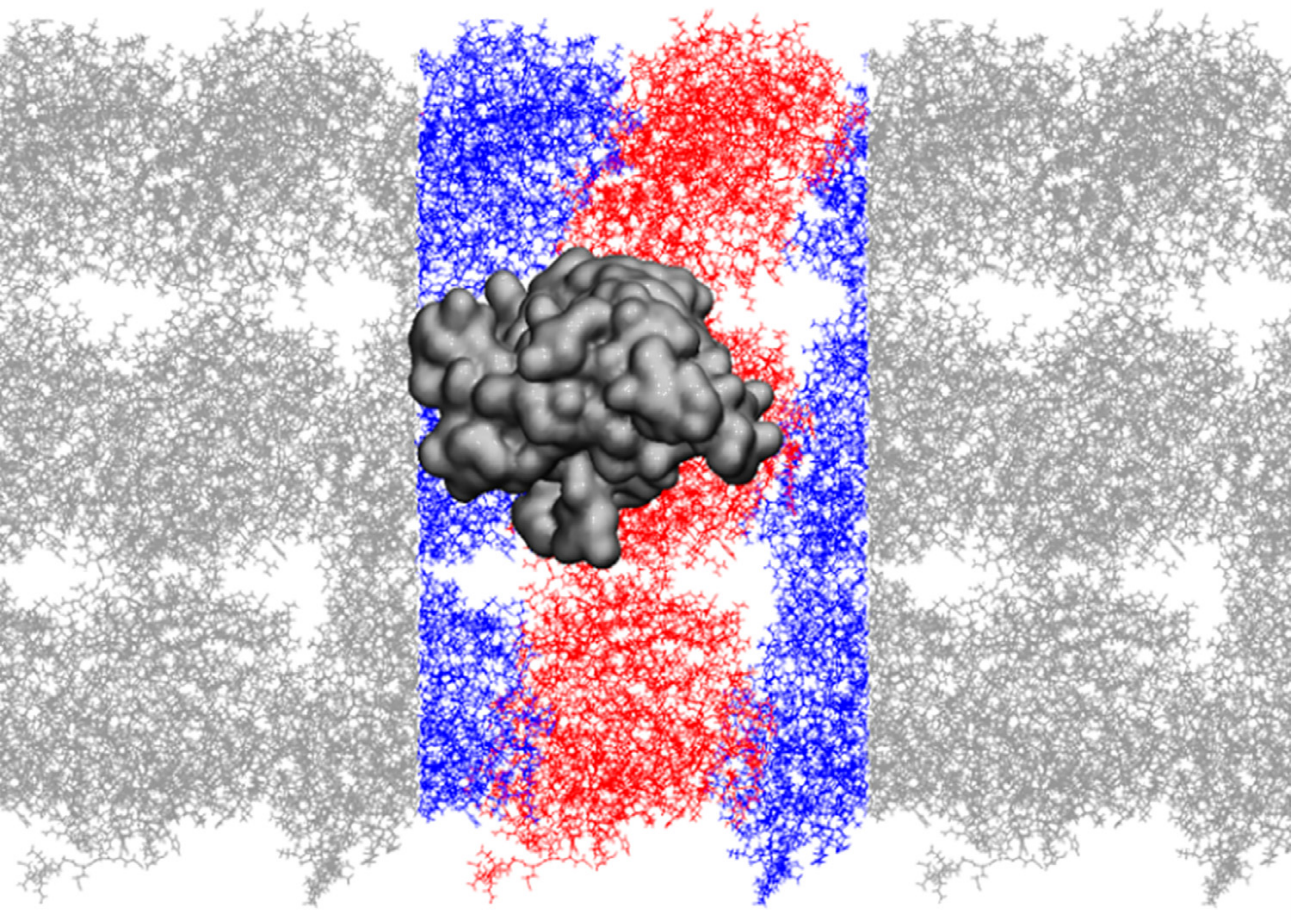
Molecular system simulated (water or ions not showed here): kinesin motor domain (metalic dark grey) docked on heterodimer of tubulin (red – beta tubulin, blue – alpha tubulin) with tubulins from two neighboring microtubule protofilaments. The system is repeated with periodic boundary conditions.

The Fig. 1 shows the structure of the molecular system simulated: a kinesin motor domain (in a metallic dark grey color), which has been docked onto a heterodimer composed of both alpha (blue) and beta (red) tubulins. The tubulins are from two adjacent microtubule protofilaments. The system has been replicated with periodic boundary conditions. The data presented in this paper are raw trajectories (.xtc files) containing positions of all atoms of the molecular system exposed to variety of electric field conditions. Additionally, the files (.tpr) containing the definition of the molecular system are also provided. A .tpr file is a GROMACS file which the necessary information to execute a molecular simulation, such as the system topology, force field parameters, and the initial atomic coordinates. It also contains the simulation box dimensions and all other simulation parameters.

The Table 1 contains information about all the raw data files from molecular dynamics simulations trajectory both for a smaller simulation box and big simulation box, which was used only for the calculation of the potential of mean force in [1]. Each line contains the file name, the electric field direction, the electric field intensity (MV/m), the number of trajectory files, and comments. The first line is X100 and it indicates that the file name is X100, the electric field direction is X, the electric field intensity is 100 MV/m, there are 40 trajectory files. The second line is Xrev100 and it indicates that the file name is Xrev100, the electric field direction is -X, the electric field intensity is 100 MV/m, there are 10 trajectory files. The third line is Z100 and it indicates that the file name is Z100, the electric field direction is Z, the electric field intensity is 100 MV/m, there are 10 trajectory files. The fourth line is Zrev100 and it indicates that the file name is Zrev100, the electric field direction is -Z, the electric field intensity is 100 MV/m, there are 10 trajectory files. The fifth line is X75-p1 and it indicates that the file name is X75, the archive is the first part, the electric field direction is X, the electric field intensity is 75 MV/m, there are 18 trajectory files. This pattern continues for the remaining lines. The last lines are for prod_NF.xtc and system.tpr and these indicate that the file name is prod_NF.xtc and system.tpr, there is zero electric field, there is 1 trajectory file for NF and no trajectory files for system.tpr, and the comments indicate that system.tpr is a small box system. The BigBox-100.xtc is a trajectory with 100 MV/m, X direction electric field and BigBox-system.tpr is a big box system, see Section 4 for more details. The Table 1 also includes the value of the median detachment time of kinesin from tubulin in nanoseconds for each electric field configuration.

**Table 1:** List of raw data files available. Trajectory files in archives are of .xtc type.

archive / file name	Electric field direction	Electric field intensity (MV/m)	Number of trajectory files	Median detachment time of kinesin from tubulin (ns)	comments
**small box files**					
X100	X	100	40	4.4	
Xrev100	-X	100	10	6.1	
Z100	Z	100	10	13	
Zrev100	-Z	100	10	16.6	
X75-p1	X	75	18	8.3	
X75-p2	X	75	12	–	
X50-p1	X	50	12	28.1	
X50-p2	X	50	8	–	
X30-p1	X	30	3	118.2	
X30-p2	X	30	3	–	
X30-p3	X	30	2	–	
X30-p4	X	30	2	–	
prod_NF.xtc	–	0	1	–	
system.tpr	–	–	–	–	small box system
**big box files**					
BigBox-100.xtc	X	100	1	–	
BigBox-system.tpr	–	100	1	–	big box system

## 4 Experimental Design, Materials and Methods

### 4.1 Molecular structure and molecular dynamics simulations

With GROMACS 2018 version software we prepare our system consisting of three tubulin heterodimers (including C-terminus) [8] with guanosine triphosphate (GTP), kinesin with adenosine diphosphate (ADP) [13,14]. The three tubulins are positioned as if they were placed in the microtubule B-lattice [8]. The tubulin structure contains GTP and originates from PDB structure ID 3J6E. The kinesin corresponds to the kinesin-1 type (kif5b gene https://www.ncbi.nlm.nih.gov/gene/3799- present in variety of organisms including humans) its structure is identical to that in [13] – it is the kinesin model built on the basis of an ADP-bound kinesin-1 structure (PDB ID: 1BG2) with neck linker truncated at residue 325.)

For the small box simulations, there were 66653 water molecules, 456 potassium (K^+^) ions and 300 chloride (Cl^-^) ions in rectangular box (20.8×14.5×8.4 nm). For the big box, there were 178528 water molecules, 996 K^+^ ions, 840 Cl^-^ ions, box dimensions 30.4 nm x 23.1 nm x 8.3 nm). The simulations employed periodic boundary conditions in a way resulting into “infinite” length tubulin strands in Z-axis of the box. See the Table 2 for the full list of number of molecules and atoms.

**Table 2:** Number of molecules and atoms in the molecular dynamics simulation.

Molecule	Number of Molecules	Number of Atoms
Water	66,653	199,959
K^+^	456	456
Cl^−^	300	300
α-Tubulin	3	20,739 (6,913 × 3)
β-Tubulin	3	20,526 (6,842 × 3)
Kinesin	1	5,068
ADP	1	39
GTP	6	264 (44 × 6)
Mg^2+^	7	7
Total	—	247,358

We have used CHARMM36(mar2019) [15] force field parameters for all atoms. After the minimization of potential energy of our system by steepest descent algorithm, we follow this procedure for the equilibration of the system:

1. 100ps (2fs step), NVT - Berendsen (300K), All-bonds constrained with LINCS algorithm, initial velocities of all particles assigned from Maxwell-Boltzmann (M-B) distribution corresponding to 300K, all heavy atoms positions restrained
2. 200ps (2fs step), NpT - Velocity rescale (300K), Parrinello-Rahman (1bar), All-bonds constrained with LINCS algorithm, all heavy atoms positions restrained, velocities of all particles inherited from step 1
3. 10ps (1fs step), NVT - Nose-Hoover (150K, 0.05 ps coupling period), All-bonds constrained with LINCS algorithm, volume of the box inherited from step 2, velocities generated
4. 300ps (2fs step), NVT - Nose-Hoover (300K, 0.5 ps coupling period), All-bonds constrained with LINCS algorithm, volume of the box and velocities of all particles inherited from step 3
5. 60 ns (2fs step), NVT - Nose-Hoover (300K, 1 ps coupling period), H-bonds constrained with LINCS algorithm, volume of the box and velocities of all particles inherited from step 4

We have used the last step of equilibration phase as a starting point for our production runs. To provide different starting conditions (coordinates and velocities) for each run, each run started by 200 ps pre-run in NVT - Nose-Hoover (300 K), H-bonds constrained and randomly assigned velocities from M-B distribution. This was followed by main phase of production run with applied external electric field with following parameters: leap-frog algorithm, 2 fs integration step, H-bonds constrained, Temperature coupled by Nose-Hoover thermostat (300 K, 1 ps period for coupling), No pressure coupling, Short-range forces by cut-off at 1.1 nm, long-range electrostatics were treated by particle mesh Ewald method. During the simulation production run, a harmonic potential with a force constant of 1,000 kJ/(mol·nm²) is employed to maintain the positions of the heavy atoms within the two outer heterodimers. This is done to reflect the binding of tubulin within the microtubule lattice. The same conditions apply for the reference run without the applied external electric field.

In the molecular system, we applied an electric field (EF) by exerting a force $\vec{F_{k}}$ on each charged atom *k* within a simulation box, where $\vec{F_{k}}=q_{k}\vec{E}$ with $q_{k}$ representing the charge of the atom and $\vec{E}$ indicating the electric field vector.

For the EF strength of 100 MV/m, we obtained 40 molecular dynamics trajectories in the X direction of EF, 10 in the -X direction, 10 in the Z direction, and 10 in the -Z direction. Additionally, we obtained 30 trajectories for the 75 MV/m EF, 20 for the 50 MV/m EF, and 10 for the 30 MV/m EF, all in the X direction. Lower EF strengths were not explored due to the excessively long simulation times required to observe the key effects we observed at the selected EF strengths. In total, the simulations spanned approximately 4.4 microseconds.

## Ethics Statement

Authors confirm that this work meets the requirements of https://www.elsevier.com/authors/policies-and-guidelines

## CRediT authorship contribution statement

**Jiří Průša:** Data curation, Formal analysis, Investigation, Methodology, Software, Visualization, Writing – original draft, Writing – review & editing. **Michal Cifra:** Conceptualization, Formal analysis, Funding acquisition, Investigation, Project administration, Resources, Supervision, Validation, Visualization, Writing – original draft, Writing – review & editing.

## Data Availability

Raw data for the paper “Molecular dynamics simulation trajectories dataset of a kinesin on tubulin heterodimers in electric field” A2103 (Original data) (ASEP Repository - Repository of Czech Academy of Sciences)
